# Prophylactic perioperative cefuroxime levels in plasma and adipose tissue at the time of caesarean section (C-LACE): a protocol for a pilot experimental, prospective study with non-probability sampling to determine interpatient variability

**DOI:** 10.1186/s40814-021-00794-3

**Published:** 2021-02-18

**Authors:** Hanadi H. Alrammaal, Hannah K. Batchelor, Hsu P. Chong, Victoria Hodgetts Morton, R. Katie Morris

**Affiliations:** 1 School of Pharmacy, Institute of Clinical Sciences, Robert Aitken Building, University of Birmingham, Edgbaston, Birmingham, B15 2TT UK; 2 Clinical Pharmacy Department, College of Pharmacy, Umm Al-Qura University, Makkah, Makkah Province Saudi Arabia; 3 Strathclyde Institute of Pharmacy and Biomedical Sciences, University of Strathclyde, 161 Cathedral Street, Glasgow, G4 0RE UK; 4 Rosie Maternity Hospital, Robinson Way, Cambridge, CB2 0SQ UK; 5 Department of Fetal and Maternal Medicine, Birmingham Women’s and Children’s NHS Foundation Trust, Edgbaston, Birmingham, B15 2TG UK; 6 Institute for Metabolic and Systems Research, University of Birmingham, Birmingham, UK

**Keywords:** Caesarean section, Cefuroxime, Pharmacokinetics, Obese, Pregnant women, Surgical site infection

## Abstract

**Background:**

The aim of the C-LACE study is to measure cefuroxime concentration in plasma and adipose tissue of non-obese and obese pregnant women undergoing caesarean section.

**Methods:**

This study plans to compare maternal cefuroxime concentrations (plasma and adipose tissue), at the time of skin incision and time of skin closure during a caesarean section from non-obese (body mass index BMI < 30 kg/m^2^) and obese (BMI ≥ 30 kg/m^2^) pregnant women. The incidence of post-surgical site infection will also be measured. At least 15 participants are required for each arm (non-obese vs obese) with a total of 30 participants. The study participants will be followed up between 30 and 40 days post-caesarean section to record details of any post-caesarean surgical infection to explore correlations between BMI, measured cefuroxime concentrations and post-caesarean infection rates.

**Discussion:**

This pilot study will allow the development of a model testing the inter-patient variability in plasma and adipose tissue concentrations of cefuroxime. The results will facilitate the development of a larger study to determine whether differences in cefuroxime plasma and tissue concentration in obese and non-obese women can support the development of a physiologically based pharmacokinetic model. This model can then be used to propose dosing adjustments that can be used in a further trial to optimise cefuroxime dosing for women undergoing caesarean section.

**Trial registration:**

ISRCTN Registry, ISRCTN17527512. Registered on 26 October 2020

**Supplementary Information:**

The online version contains supplementary material available at 10.1186/s40814-021-00794-3.

## Administrative information

This protocol follows the SPIRIT checklist (items of the checklist are numbered in curly brackets).

## Background {6a}

The prevalence of obesity in pregnant women in England at the time of booking a caesarean section (CS) has been reported to be 16% [1, 2]. One complication of obesity in pregnancy relates to the requirement for operative delivery, i.e. CS. The rate of CS in an obese (Body Mass Index BMI ≥ 30 kg/m^2^) pregnant population is 33.8% [3]. The rate of post-CS infection is higher amongst obese pregnant women compared to those who are not obese; the adjusted odds ratio of surgical site infection in an obese population was reported to be 2.41 (95% CI 1.73–3.37) [4]. Prophylactic single dose perioperative intravenous (IV) antibiotic is recommended for CS; and several studies have examined the use of alternative antibiotics, with cefazolin being the most commonly used in the USA and cefuroxime in the UK. Numerous studies have focussed on the use of cefazolin in obese patients undergoing CS to gain an understanding of optimal dosing and clinical outcomes [5]. Several studies proposed an increased dose of cefazolin (3 g) compared to the standard dose (2 g) for obese pregnant women to achieve sufficient antibiotic levels during CS [6–8]. It was postulated by Holt et al. (in 1994) that doses of cefuroxime up to 1500 mg may not achieve sufficient minimum inhibitory concentration (MIC) for most of the common bacteria in maternal blood during CS [9]. Sub-therapeutic antibiotic concentration is a crucial factor in global bacterial resistance. It is essential that the therapeutic concentration of antibiotic is achieved in both maternal blood and at the site of incision during CS for effective therapy. No study to date has measured cefuroxime concentrations in blood and adipose tissue in obese pregnant women undergoing CS [10]. Thus, a study to explore the drug concentration in this population at the time of CS is essential. This study will analyse cefuroxime concentration in plasma and adipose tissue samples to accurately measure levels achieved in non-obese and obese pregnant women undergoing CS to confirm if therapeutical cefuroxime levels are reached in plasma and adipose tissue. Further, to use the measured cefuroxime concentrations to verify a physiologically based pharmacokinetic (PBPK) model of cefuroxime in CS to explore the impact of (i) dose and (ii) BMI post-administration of cefuroxime on both blood and tissue cefuroxime concentrations in an obese population and suggest a dose adjustment if required and applicable.

### Objectives {7}

The main objective of conducting this pilot and feasibility study is to assess the process and protocol of recruiting patients, sample collection, follow-up and procedure of C-LACE. Also to gain an understanding of cefuroxime concentrations in normal-body weight pregnant vs. obese pregnant women. The primary objective is to measure the concentration of cefuroxime in the collected blood and tissue samples using an appropriate analytical method and to assess whether cefuroxime administered provides concentrations above the MIC in both the blood and adipose tissue for all patients regardless of their BMI. Additionally, to assess whether there are differences in cefuroxime concentrations from obese vs non-obese women.

### Trial design {8}

A single-centre experimental, prospective pilot study with non-probability sampling.

## Methods/design

### Aims and methodologies

To measure cefuroxime concentration in plasma and adipose tissue (within the incision) at time of skin incision and skin closure in obese and non-obese pregnant patients requiring CS. Moreover, to explore the relationship between the dose of cefuroxime administered and concentration measured in both blood and adipose tissue with respect to BMI of the patient. To measure the incidence of post-CS infections in obese and non-obese pregnant women at 30–40 days post-CS; and to assess if the rate of infection is correlated with BMI, dose administered and cefuroxime blood or adipose tissue concentrations.

Cefuroxime is administrated to pregnant women undergoing CS as per Birmingham Women’s Hospital protocol; i.e. IV infusion 30–60 min before skin incision.

### Outcomes {12}

Outcomes of C-LACE pilot and feasibility study are AS FOLLOWS:

- Patients’ recruitment rate
- Collecting samples strategies
- Analytical method for sample analysis
- Data collection and forms completion
- Follow-up and participants’ retention

### Part A: Cefuroxime levels in plasma and adipose tissue

The minimum inhibitory concentration is not harmonised between guidelines; thus, the time above the minimum inhibitory concentration (T>MIC) will be reported for MIC values of 1, 4 and 8 mg/L [5, 11] as these values correlate to the most common bacteria involves in post-CS surgical infections (Table 1).

**Table 1:** Most common bacteria causative of infection post-caesarean section and the minimum inhibitory concentration range/breakpoint

	Microorganism	MIC range/breakpoint (mg/L) [5, 11–13]
Gram-negative bacteria	*Escherichia coli*	0.001–8
	*Haemophilus influenzae*	2
	*Klebsiella oxytoca*	8
	*Proteus mirabilis*	0.001–8
	*Ureaplasma urealyticum*	ND
Gram-positive bacteria	*Enterococcus faecalis*	ND
	*Staphylococcus aureus*	4
	*Staphylococcus epidermidis*	0.25–4
	*Streptococcus pneumoniae*	1
Other organisms	*Raoultella ornithinolytica*	0.001–8
	*Morganella morganii*	ND

*ND* Not determined

### Part B: Surgical site infection

The rate of surgical site infection will be reported in non-obese and obese pregnant women undergoing CS. This study will follow the definition of surgical infection as per the definitions set out by the US Centre’s for Disease Control and Prevention (Centre’s for Disease Control and Prevention 2019) [14].

### Target population

The target population are non-obese and obese pregnant women undergoing elective CS at Birmingham Women’s Hospital.

### Eligibility criteria {10}

#### Inclusion criteria

- Pregnant women 18 to 50 years old, with a singleton pregnancy undergoing elective CS at 37 weeks or over.
- Participant is able to give consent and agree to sample storage.
- Participant agrees to be contacted for follow-up.
- Participant contributing to the study needs to be able to read and/or understand English.

#### Exclusion criteria

- Multiple pregnancy
- Previous CS that resulted in a surgical site infection
- Emergency CS
- Any non-elective CS including those requiring early delivery without threat to maternal or foetal health that were not previously planned.
- Body mass index (BMI) less than 18 kg/m^2^ or greater than or equal to 45 kg/m^2^ (at time of first pregnancy appointment and at time of delivery).
- Currently enrolled in a randomized controlled trial for an intervention to reduce post-operative surgical site infection
- Diabetes (type 1, type 2 or gestational)
- Hypertension
- Renal disease
- Cardiovascular disease (e.g. maternal structural cardiac disease)
- Liver disease
- Inflammatory bowel disease (e.g. Crohn’s disease or ulcerative colitis)
- Cephalosporin or penicillin allergy
- Administration of antibiotic within 1 week prior to delivery
- Suspected pre-existing infection (including chorioamnionitis)
- Autoimmune disease (e.g. systemic lupus erythematosus, rheumatoid arthritis)
- Chronic use of corticosteroid
- History of wound breakdown in an abdominal surgery
- Prior laparotomy for any indication (e.g. previous ovarian cystectomy or previous bowel surgery)

### Setting {9}

This single-centre study will take place at Birmingham Women’s Hospital which is a large teaching hospital that is part of Birmingham Women’s and Children’s Hospital NHS Foundation Trust.

### Participant selection and enrolment

Any patient (18–50 years) who requires a CS as part of their clinical care will be screened for inclusion. The potential participants will be identified using hospital clinical lists, where lists relate to a pre-booked CS a Good Clinical Practice (GCP) trained research midwife who is a member of the clinical care team will assess the eligibility of patients to participate in the study according to inclusion/exclusion criteria using screening form. The woman will then be provided with the participant information sheet at this meeting by the research midwife. In addition, if a pregnant woman is booked for CS at a meeting they may be informed about the study by a research midwife or their clinical care team and provided with a participant information sheet. The participants will receive information about the study well in advance (at prior clinic appointments) and at least 24 hours prior to CS so that all participants have sufficient time to consider taking part.

This study plans to compare data from obese (BMI ≥ 30 kg/m^2^) and non-obese women (BMI < 30 kg/m^2^) at the time of booking (10–14 weeks of pregnancy). At least 15 participants are required for each arm with a total of 30 participants. The study sequence is summarised in Table 2.

**Table 2:** Summary of the C-LACE study sequence and methodology

Timing	Activity
Caesarean section booking clinic	Clinic list will be reviewed for eligible participants using the screening form
On the day of caesarean section	A Venflon cannula will be inserted into the woman as part of the research process to enable withdrawal of the required blood samples without the need for additional needles. This second access point will ensure that the blood sampling is minimally disruptive to the CS procedure.
At 30–40 days post-caesarean section	Participant’s medical file and notes will be reviewed by the research midwife to identify any post-surgical site infection which will be recorded on the 6-week outcome form. Research midwife will attempt to contact the participant 3 times between 30 and 40 days post-CS to complete the follow-up interview form.
Samples analysis	Blood and adipose tissue will be analysed by LC-MS/MS [15]

*GCP* Good Clinical Practice, *CS* Caesarean section, *LC-MS/MS* liquid Chromatography with tandem mass spectrometry

### Screening for eligibility and recruitment {15}

A GCP trained research midwife will assess the eligibility of patients to participate in the study according to inclusion/exclusion criteria using screening form.

### Ineligible and non-recruited participants

Certain ineligible are stated in the exclusion criteria. Research midwives/clinician will review and reassess each potential participant and confirm eligibility of patients to participate in the study at the day of CS.

### Patient consent {26a}

A suitably qualified research midwife or doctor who is also part of the clinical care team will introduce the study to the woman at their pre-operative assessment. A participant information sheet (PIS) will be provided to facilitate this process. Investigators will ensure that they adequately explain the aim, anticipated benefits and potential hazards of taking part in the trial to the woman. They will also stress that participation is voluntary and that the woman is free to refuse to take part and may withdraw from the trial at any time. The woman will be given adequate time to read the PIS and to discuss their potential participation and be given the opportunity to ask questions. If the woman expresses an interest in participating in the trial, they will be asked to sign and date the latest version of the informed consent form at least 24 h prior to CS. The woman will be asked for her consent to withdraw 3 blood samples (with a maximum volume of 10 mL blood per sample) and 2 fat tissues and for collection of outcome data from their medical records and a telephone interview at 30–40 days post-natal that will take approximately 5–15 min.

### Withdrawal of study participants

Each participant has the right to withdraw from the study at any time and this will not affect their clinical care. In addition, the investigator may discontinue a participant from the study at any time if the investigator considers it necessary for any reason including the following:

- Ineligibility (either arising during the study or retrospectively having been overlooked at screening)
- Withdrawal of consent

Withdrawal from the study will result in the exclusion of the data for that participant from analysis; provided that the withdrawal occurs within 3 months of data collection.

### Study assessments {13}

The study assessment and paperwork associated are summarised in Table 3 (forms are in the online supplementary file).

**Table 3:** Schedule of assessments and associated paperwork

Visit	Screening form	Consent form	Baseline form	Eligibility reassessment form	CS procedure form	Collection and labelling of samples	Discharge form	Follow-up interview form	Medical record check form
Eligibility check	×								
Consent at least 24 h pre-CS		×	×	×					
Day of CS until discharge					×	×	×		
Follow-up								×	×

### Data collection methods {18a}

Background information will be collected from each participant using the baseline form. This information is required for interpretation of the data and dissemination.

Following the collection of the data from all forms (screening, consent, baseline, eligibility reassessment, operation, discharge, follow-up interview, 6-week record check) will be filled, signed and shared via the secure system; this system can be accessed at the university and the hospital and ensures the security of data, any data, entered will be anonymised.

The study has two parts: part A, which is blood and adipose tissue sampling for cefuroxime concentrations; part B, is a follow-up of patients’ post-partum at between 30 and 40 days to record the incidence of post-surgical infection.

#### Part A: Sample collection at caesarean section

The clinical care team will record the time of cefuroxime administration, dose administered, type of anaesthesia used, blood loss and other relevant clinical information. Five samples will be collected: 1 blood sample at time of skin incision, 1 blood sample at time of skin closure, 1 blood sample at recovery room (< 3 h following CS), 1 adipose tissue sample (approximately 1 cm from the skin in the middle of the Pfannesnstiel or vertical midline incision) at skin incision and one adipose tissue sample (from a similar location to sample 1) just prior to skin closure.

### Blood sample and adipose tissue analysis

The concentration of cefuroxime in blood and adipose tissue will be quantified using standard laboratory methods [15].

#### Part B: Follow-up for post-surgical infection

At between 30 and 40 days post-CS, the study participants will be contacted by telephone by a research nurse/midwife from the research team and a review will be made to record any type of post-surgical infection. The follow-up interview form will be used to record the details of any infection reported. If the participant attended to the hospital for a post-natal appointment at 30–40 days, the interview can be conducted at that time to minimise the burden to the participant.

### Plans to promote participant retention and complete follow-up {18b}

Patient medical notes will be reviewed by a member of the clinical care team using the 30–40 days outcome form to identify whether there has been a reported post-surgical infection. Post-surgical infection is defined as per the definitions set out by the US Centre’s for Disease Control and Prevention [14].

### Data management {19} and confidentiality {27}

All participants will be given a study ID upon recruitment to the study. A spreadsheet that links study ID to patient ID will be held at Birmingham Women’s and Children’s Hospital NHS Foundation Trust under the control of the local PI. This information will be password protected and stored on an encrypted computer that conforms to the standard NHS security requirements.

The study ID will be used on all forms; this information provides anonymised data to the University of Birmingham that no longer has any personal identifiable data. Following collection the data from all forms will be entered on a secure system at the NHS site (undertaken by the research midwife); this system can be accessed at the university and ensures anonymity for the participants.

All processes will be carried out with respect to participant confidentiality. All participant data will be anonymised by being replaced with a study ID number prior to data sharing with the University of Birmingham. The anonymity code will only be known to the local site PI and associated research nurse/midwife team. The data will be controlled by usernames and encrypted passwords.

### Statistics {20a}, sample size {14} and study assessment

There is no predefined sample size yet sufficient numbers are required to provide an estimate of average values as well as the level of inter-patient variability. Previous work to measure the concentration of antibiotics in plasma and in tissue in obese and non-obese pregnant women following CS has used sample sizes of 14 in each arm to detect differences in adipose antibiotic concentration [16, 17]. Therefore, in this study, we plan to recruit 15 women in each arm (BMI < 30 kg/m^2^ and BMI ≥ 30 kg/m^2^). The BMI measurements will be taken from the women at their time of booking appointment which occurs between 10 and 14 weeks of pregnancy. The BMI of participants immediately prior to CS will be determined to review any major changes during their pregnancy which may affect the results, for example, a change from < 30 kg/m^2^ to an extremely obese participant at CS.

The differences in mean concentration values in plasma and adipose tissue between the obese and non-obese populations will be compared using ANOVA analysis to determine whether there are significant differences. SPSS software will be used for all statistical analysis. The rate and type of infection will be analysed. The rate of infections will be analysed in correlation with (1) dose administered, (2) BMI and (3) cefuroxime concentrations at both sites at the time of skin incision and skin closure.

#### Success criteria and assessment of C-LACE pilot and feasibility study

These criteria applied to participants consented and did not withdraw from the study at any time-point of C-LACE study:

- Patients’ recruitment rate: as per Birmingham Women’s Hospital Caesarean Section rate, recruitment of 30 patients would be achievable easily within 3 months. If this was not achieved, the protocol (specific inclusion and exclusion criteria) would be reviewed and reassessed.
- Collecting samples: this pilot and feasibility study would be considered successful if plasma and adipose tissue samples at the time of incision and time of closure and plasma sample at time of recovery were successfully collected for ≥ 75% of participant in each arm. If this was not achievable, the sampling strategies will be revised and reassessed.
- Sample analysis: this pilot and feasibility study would be considered successful if ≥ 80% of plasma and adipose tissue samples collected were successfully analysed via the analytical method described; If not, sampling handling, transferring and sample analysis protocol will be revised and reassessed.
- Data collecting: this pilot and feasibility study would be considered successful if ≥ 80% of data collecting forms was fulfilled for each participant; if not, logistics and study procedure of C-LACE study will be revised at Birmingham Women’s Hospital.
- Follow-up: the described follow-up protocol will be considered successful if participants’ retention were ≥ 80%. The participant will be considered as lost to follow-up, if the participant could not be contacted and the patients file and notes were not reviewed.

Analysis and sub-analysis of outcomes and success criteria of C-LACE pilot and feasibility study will be made to explore the reasons of unsuccessful.

### Missing data and lost to follow-up {20C}

Every attempt will be made to collect full follow-up data for all study participants; it is thus anticipated that missing data will be minimal. The main analysis will use available data only; however, the amount of missing data will be assessed, and if necessary, sensitivity analyses will be undertaken. If the participant could not be contacted, but the patients’ medical records and notes were reviewed, the participant will be considered enrolled in the follow-up. The patient will be considered as lost to follow-up, if the participant could not be contacted and the patients’ file and notes were not reviewed.

### Data monitoring {21a}

The study may be monitored or audited in accordance with the current approved protocol, GCP, relevant regulations and standard operating procedures. This study does not include blinding and patients are following the standard clinical pathway where the only intervention is the taking of adipose tissue and plasma samples therefore a data monitoring committee is not required.

### Access to data {29}

All data will be collected only after the participant freely gave written consent to process their data in accordance with GDPR (this consent in included within the consent form for the study). Direct access to shared secure system will be granted to authorised representatives from the Sponsor and host institution for monitoring and/or audit of the study to ensure compliance with regulations.

### Harms {22}

The research protocol does not expose the participants to any risks as the study does not affect their clinical treatment. The collection of additional blood samples and adipose tissue samples during CS are the only additional study-specific procedures. These additional blood samples will be taken by a clinical member of the research team trained in phlebotomy. There is a consent request for these study-specific procedures to minimise the changes to routine clinical care and reduce the burden to participants. The risks associated with this research are minimal to participants.

All participants will be contacted at 30–40 days following CS to ask about post-surgical infections; this will not affect their clinical care in any way and does not pose any risks to participants.

### Provisions for post-trial care {30}

Participants within this trial will follow standard clinical procedures with the intervention being taking plasma and adipose tissues samples thus there is no specific post-trial care. As the participants are NHS patients, indemnity is provided through the NHS schemes. The University of Birmingham provides clinical research insurance for studies that it sponsors. This insurance is provided via membership of UMAL.

### Auditing {23}

The study may be monitored, or audited in accordance with the current approved protocol, GCP, relevant regulations and standard operating procedures.

### Protocol amendments {25}

The Investigator will submit and, where necessary, obtain approval from the Health Research Authority (HRA) and Health and Care Research Wales (HCRW) for all substantial amendments to the original approved documents.

## Discussion

### Interpatient pharmacokinetic variability of cefuroxime

Due to the great variability associated with pharmacokinetic measurements, the use of PBPK modelling is a common tool to understand the distribution of drugs. Specialist software packages are available that include details on pregnant population characteristics. SimCYP software will be used to model the blood and adipose concentrations of cefuroxime and to compare this modelled data to the experimental results from the C-LACE study. It is anticipated that this clinical data coupled with PBPK models will inform any future changes to doses of cefuroxime used for CS, particularly in relation to obese women.

### Definition of the end of study

The end of study is the date at which the follow-up interview is conducted at 30–40 days following CS. This is where the follow-up ends for all participants.

### Patient and public involvement

A similar study conducted by Victoria Hodgetts Morton and Katie Morris (PREPS trial) involved the public and patients; those involved were supportive of any research undertaken to minimise the risks of post-surgical infections following CS [18]. Informal discussions about this study with women who have had CS have all been positive as the burden of participation is minimised where possible.

### Ethics and dissemination {31a-b-c}

Results of this trial will be submitted for publication in a peer-reviewed journal. The manuscript will be prepared by HHA and HKB with contributions from the local PI (RKM) and the local researcher (VHM) and authorship will be determined by mutual agreement. Authors must acknowledge that the trial was performed with the support of the Saudi Arabian Government. Currently, there are no plans for granting public access to the full protocol, participant-level dataset and statistical code.

### Provenance and peer review

The protocol has been peer-reviewed within the research team and by the research and governance team at the University of Birmingham as part of the agreement to sponsor the study and by the University of Birmingham’s Clinical Research Compliance Team (CRCT). Further, this protocol has been reviewed and approved by HRA and HCRW.

### Trial status

Ethical approval is granted (C-LACE protocol v0.4, 02 January 2020); the anticipated date of recruitment was 1st of September 2020; requirement expected to be completed on 1st of December 2020). This study was delayed due to COVID-19 epidemic.

## Supplementary Information

**Additional file 1.**

**Additional file 2.** C-LACE Screening Form.

**Additional file 3.** SPIRIT 2013 Checklist: Recommended items to address in a clinical trial protocol and related documents*.

## Data Availability

Direct access will be granted to authorised representatives from the sponsor and host institution for monitoring and/or audit of the study to ensure compliance with regulations. Access to the data retrieved from participants will be restricted. The clinical research team are those based at the hospital and the paperwork will not be transferred to the University of Birmingham. Following collection the data from all forms will be entered on a secure system at the NHS site (undertaken by the research midwife); this system can be accessed at the university and ensures anonymity for the participants. Researcher from the research team from the University of Birmingham will use the anonymous data to perform the data analysis.

## Abbreviations

BMI: Body mass index
CS: Caesarean section
GCP: Good clinical practice
IV: Intravenous
LC-MS/MS: Liquid chromatography with tandem mass spectrometry
MIC: Minimum inhibitory concentration
PBPK: Physiologically based pharmacokinetic
PIS: Participant information sheet
T>MIC: Time above the minimum inhibitory concentration
