# Supplementary material for: Prophylactic perioperative cefuroxime levels in plasma and adipose tissue at the time of caesarean section (C-LACE): a protocol for a pilot experimental, prospective study with non-probability sampling to determine interpatient variability
Source: Pilot Feasibility Stud. 2021 Feb 18;7:54. doi: 10.1186/s40814-021-00794-3 (PMC7890388; doi:10.1186/s40814-021-00794-3)
Supplement: Supplementary file 1 — Additional file 1. [file 40814_2021_794_MOESM1_ESM.docx]

**CONSENT FORM for the participant** {32}

**C-LACE:** **C**efuroxime **L**evels **A**fter **C**aesarean **S**ection

|  |  |  |  |  |
| --- | --- | --- | --- | --- |

Participant Study ID Number:

Please initial box

| 1 | I confirm that I have read and understood the Participant information sheet (v0.2 16-09-2019) for the above study. |  |
| --- | --- | --- |
| 2 | I have had the opportunity to ask questions and all my questions have been answered to my satisfaction. |  |
| 3 | I understand my participation is voluntary and that I am free to withdraw at any time, without giving any reason, without my medical care or legal rights being affected. |  |
| 4 | I understand that sections of my medical notes may be reviewed by responsible individuals from Birmingham Women’s Hospital, University of Birmingham as Sponsor or regulatory authorities, where it is relevant to my taking part in research.  I give permission for these individuals to have access to these records. |  |
| 5 | I Agree to my medical records being accessed to share the following information about me (in an anonymised format) with the University of Birmingham (ethnicity; height; weight; site from where sample(s) removed; Caesarean Section operative information; follow up information). |  |
| 6 | I agree to the collection of blood samples and tissue samples taken during Caesarean Section and for these samples to be transported and stored at the University of Birmingham for use in this research project. |  |
| 7 | I agree to being contacted by a research midwife from Birmingham Women’s Hospital by telephone 30-40 days after my Caesarean Section and being asked questions about whether I have had an infection as a result of my C-section. |  |
| 8 | The procedures regarding confidentiality have been clearly explained to me (e.g. anonymization of data, etc.). |  |
| 9 | I acknowledge that the data will be stored at the University of Birmingham for period of 10 years and Birmingham Women’s Hospital for 25 years in line with local Hospital policy, with the access restricted only to the research team. |  |
| 10 | The use of the data in research, publications has been explained to me. I will not be mentioned by name in any publications as the data will be coded and non-identifiable. |  |
| 11 | I agree to my personal information being collected and processed for the purpose of the study in accordance with General Data Protection Regulation (GDPR). |  |
| 12 | I agree to participate in this study. |  |
| 13 | OPTIONAL: I agree to any remaining tissue to be used in other research at the University of Birmingham with the relevant ethical approval. I understand that this will be a donation from me to the University of Birmingham. |  |

Participant name:_____________________________________________

Signature: Date:

Name of person taking consent:

Signature: Date:

*Original copy for the study site file, one copy for the medical records and one for the participant*

IRAS# 244803 *Informed Consent Form v0.4 18^th^ December 2019*

## Sample collection at C-Section {33}

The clinical care team will use the following forms: Operation Form and Discharge Form to record the time of cefuroxime administration, dose administered, type of anaesthesia used, blood loss and other information (see documents/appendixes). Five samples will be collected: 1 blood sample at time of skin incision, 1 blood sample at time of skin closure, 1 blood sample at recovery room, 1 adipose tissue sample (approximately 1 cm from the skin in the middle of the Pfannesnstiel or vertical midline incision) at skin incision and one adipose tissue sample just prior to skin closure.

The first sample of blood will be taken at time of skin incision. After the operation, take another blood sample will be collected as close to the time of delivery as possible and the final blood sample will be taken in the recovery room. The samples will either be taken from a cannula inserted in the participant’s hand via a cannula, or taken by a very small ‘butterfly’ needle from the participant’s hand or foot. If the samples are taken from their foot this will be numb from the anaesthetic and will not hurt. The research team will discuss with the participant how best to take these samples in line with their personal preferences.

Just before your baby is delivered, we will ask your doctor to remove a small amount of the fat from under the skin on your tummy. This will be a very small amount (about the size of a 50p coin). During your C-section, you will have an anaesthetic so you should not feel any pain. Your doctor will deliver the baby and placenta then stich the womb. Just before your skin is stitched, your doctor will remove a second 50p sized sample of fat. Taking these small fat samples will take less than a minute each so the overall procedure is only a maximum of two minutes longer than without the fat samples.

The securely sealed specimen sample tube will be labelled with the participant identification number which will correspond to the details held on the background information (Sample Label). These samples will be stored refrigerated as soon after collection as possible by the laboratories at Birmingham Women’s and Children’s Hospital NHS Foundation Trust and all samples will be couriered within a cool box (on ice) to the University of Birmingham (using an approved courier for transport of human samples). Prior to any human tissue transport, all human tissues will be packed into a sealed container, and labelled with the following information: Human Tissue Sample, Sample Reference Number, Tissue type, details of medium/ preservative, date of packing.

Upon arrival at the University the samples will be handled according to the University of Birmingham Health and Safety Guidance (GUIDANCE/26/WHBFT/14) and in accordance with the HTA and local procedures. Samples will be stored at the University of Birmingham in dedicated locked areas of -80°C freezers that are suitable for storage of human samples.

At the University of Birmingham, the process of characterisation will be performed within a laboratory settings on batches of samples. SOPs are in place for subsequent processing of samples. The work at the University of Birmingham will be conducted within the Pharmaceutics Laboratory in the Sir Robert Aitken building. The facilities are HTA compliant.

## Sample Storage and Destruction

Local storage of samples at Birmingham Women’s Hospital will be managed by the Laboratory R&D team where samples will be refrigerated upon receipt and then packaged appropriately for transfer to the University of Birmingham using an approved courier.

All human tissue will be packed into a sealed container prior to transport and labelled with the following information:

**Human Tissue Sample**

Sample Reference Number:

Tissue Type:

Details of medium/preservative if applicable: Note that no preservative will be used for these samples.

Date of packing:

UN 3373 warning label

The container will then be packed into a box and labelled with an approved human tissue sample hazard warning label – UN 3373.

Samples will be stored at the University of Birmingham in appropriate freezer boxes within a -80°C locked freezer in compliance with the HTA legislation. Each freezer box will be labelled with: (i) the name of the researcher; (ii) the PI’s name; (iii) the study name; (iv) the study ethics reference number; (v) the date the trial began; (vi) the sample type in the box; (vii) the latest date by which the contents of the box must be destroyed/disposed of. Each sample will be labelled with the study identification number (which will not include any personal details of the participant).

The research team have requested consent to store any remaining tissue that is left over after this study to use for other research at the University of Birmingham. This further research will require approval from a research ethics committee. If no further research is planned and the samples need to be discarded this will be undertaken by placing samples into double autoclave bags and sent for autoclaving. Autoclaved waste will then be placed into yellow clinical waste bags and marked for incineration.

## Sample Handling in the laboratory

Human samples will be handled according to the University of Birmingham Health and Safety Guidance (GUIDANCE/26/WHBFT/14) and in accordance with the HTA and local procedures. Samples will be stored at the University of Birmingham in dedicated locked areas of -80°C freezers that are suitable for storage of human samples.

University of Birmingham Health and Safety Policy (UHSP/9/BS/14) requires that risk assessment of all work with biological materials must be carried out in advance of work commencing. This work was submitted to the University of Birmingham Advisory Group for the Control of Biological Hazards “Measuring cefuroxime concentration in human plasma and adipose tissue” - Ref: AG 16_03 (approved in May 2018).

All work will be conducted within ADCP level 2 containment. As per standard laboratory practice the researcher will wear laboratory coat and nitrile gloves when handling human samples.

Chemgene HLD4L for disinfection validated to BS EN 14476:2005 will be used to clean all apparatus both prior to and following contact with the human samples used within this study. This will be diluted to 1:20 (with deionised water) to ensure that there is no risk of contamination due to contact with human samples.

Human blood and tissue will be kept in-80°C freezers, which will be located in a room with limited access to authorised staff. These freezers are specified for human tissue storage only (not animal origin).

## Methodology for characterisation of samples collected

The concentration of cefuroxime in blood and adipose tissue will be quantified using standard laboratory methods. Full methodology is available as SOPs within the pharmacy research laboratory.
