# Supplementary material for: Prophylactic perioperative cefuroxime levels in plasma and adipose tissue at the time of caesarean section (C-LACE): a protocol for a pilot experimental, prospective study with non-probability sampling to determine interpatient variability
Source: Pilot Feasibility Stud. 2021 Feb 18;7:54. doi: 10.1186/s40814-021-00794-3 (PMC7890388; doi:10.1186/s40814-021-00794-3)
Supplement: Supplementary file 2 — Additional file 2. C-LACE Screening Form. [file 40814_2021_794_MOESM2_ESM.docx]

C-LACE ScreeningForm

| **Section 1 - Details of the Participant** | | | | | | | | | | | | | |
| --- | --- | --- | --- | --- | --- | --- | --- | --- | --- | --- | --- | --- | --- |
| \|  \|  \|  \| \| --- \| --- \| --- \|   Initials of the woman: *First, Middle, Last* | Date of Birth: | *e.g. 31-JAN-1997* | **D** | **D** | - | **M** | **M** | **M** | - | **Y** | **Y** | **Y** | **Y** |

| Section 2 - Screenings | | |
| --- | --- | --- |
| Inclusion Checklist | | |
| Age of woman is between 18 years to 50 years? *Tick one* | No | Yes |
| With a singleton pregnancy? *Tick one* | No | Yes |
| Having an elective Caesarean Section? *Tick one* | No | Yes |
| Caesarean scheduled for at 37 weeks or over (≥37weeks)? *Tick one* | No | Yes |
| With no known allergy to cefuroxime, cephalosporin or penicillin? *Tick one* | No | Yes |
| Exclusion Checklist | | |
| Previously had an infection following a Caesarean Section? *Tick one* | No | Yes |
| Body Mass Index (BMI) less than18 kg/m^2^ at first pregnancy appointment? *Tick one* | No | Yes |
| Body Mass Index (BMI) greater than or equal to 45kg/m^2^ at first pregnancy appointment?  *Tick one* | No | Yes |
| Currently enrolled in an RCT for an intervention to reduce post-operative surgical site infection? *Tick one* | No | Yes |
| Has Diabetes (type 1, Type 2 or gestation)? *Tick one* | No | Yes |
| Diagnosed with Hypertension? *Tick one* | No | Yes |
| Diagnosed with renal disease?  *Tick one* | No | Yes |
| Has a Cardiovascular Disease (eg. Maternal structural cardiac disease)? *Tick one* | No | Yes |
| Has liver disease? *Tick one* | No | Yes |
| Has inflammatory bowel disease (eg Crohn’s disease or ulcerative colitis)? *Tick one* | No | Yes |
| Prior laparotomy for any indication (eg. Previous ovarian cystectomy or bowel surgery)? *Tick one* | No | Yes |
| Suspected pre-existing infection (including Chorioamnionitis)? *Tick one* | No | Yes |
| Has autoimmune disease (eg SLE, Rheumatoid arthritis)? *Tick one* | No | Yes |
| Chronic use of corticosteroid? *Tick one* | No | Yes |
| History of wound breakdown in an abdominal surgery? *Tick one* | No | Yes |
| If this form was completed more than 1 week pre CS please do not answer this question: Administration of antibiotic within 1 week prior to delivery? | No | Yes |
| Please note: if any of the shaded boxes above are ticked, then the woman is ineligible to take part in this study. Please sign and date the form. If the women is eligible then please provide the participant information sheet to the woman. | | |
| Does the woman wish to take part in C-LACE study? | No | Yes |

| **Section 3 - Form Completion Details** | | |
| --- | --- | --- |
| Full name of person completing form: | Signature of person completing form: | Date:  **D D** - **M M M** - **Y Y Y Y** |

C-LACE Baseline Form

| Section 1 - Participant Details |
| --- |
| Participant Trial No:   \|  \|  \|  \|  \|  \| \| --- \| --- \| --- \| --- \| --- \| |

| Section 2 - Baseline Demographics | | |
| --- | --- | --- |
| Booking BMI: **.** kg/m2  *Booking BMI: BMI at time of booking CS* | Booking Weight: **.** kg  *Booking weight: BMI at time of booking CS* | Height: cm |
| Ethnicity: *Tick one*  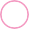 British European 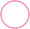 Irish European 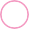 East European 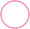 North European 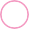 South European 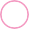 West European 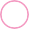 North African 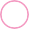 Sub-Saharan African 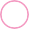 Middle Eastern 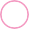 Indian 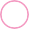 Pakistani 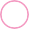 Bangladeshi 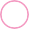 Chinese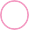 Other Far East 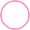 South East Asia 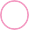 Caribbean 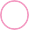 Other 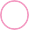 Declined to give information | | |

| Section 3 - Pre-pregnancy Medical Conditions and Pregnancy History | |
| --- | --- |
| Please tick NO if the conditions do not apply. | |
| What is the woman's parity? *e.g. 01, 02, etc.* | Number of previous caesarean sections? *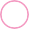* 0 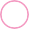 1 *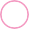* 2 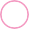 3 *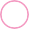* ≥4 |
| Has the woman ever had any other open abdominal surgery (excluding caesarean section)? *Tick one 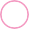* No 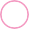 Yes | |
| If **YES**, please specify: | |

| Section 4 - This Pregnancy |
| --- |
| Estimated Date of Delivery: *e.g. 31-JAN-2019* **D D** - **M M M** - **Y Y Y Y** |

| Section 5 - Smoking, Drugs & Alcohol Use | | | |
| --- | --- | --- | --- |
| Was the woman an **ongoing** smoker at **booking** in this pregnancy? *Tick one* | | No 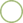 | Yes 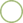 |
| Has the woman used non prescribed recreational drugs in this pregnancy (e.g. cocaine)? *Tick one* | | 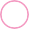 No | 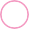 Yes |
| If **YES**, please specify | | | |
| Has the woman consumed alcohol in this pregnancy? *Tick one 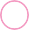* No 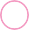 Yes | If **YES**, how many units per week? units | | |

| Section 6 - Form Completion Details | | |
| --- | --- | --- |
| Full name of person completing form: | Signature of person completing form: | Date: *e.g. 31-JAN-2017*  **D D** - **M M M** - **Y Y Y Y** |

C-LACE eligibility reassessment Form

**PLEASE complete this within 24H pre CS**

| **Section 1 - Participant Details** |
| --- |
| Participant Study ID number:   \|  \|  \|  \|  \|  \| \| --- \| --- \| --- \| --- \| --- \| |

| **Section 2 – Current BMI** | | |
| --- | --- | --- |
| Current BMI: **.** kg/m2 | Current Weight: **.** kg | Height: cm |

| Section 3 – Eligibility re-assessment | | |
| --- | --- | --- |
| Inclusion Checklist | | |
| Having an elective Caesarean Section? *Tick one* | No | Yes |
| Caesarean scheduled for at 37 weeks or over (≥37weeks)?? *Tick one* | No | Yes |
| With no known allergy to cefuroxime, cephalosporin or penicillin? *Tick one* | No | Yes |
| Exclusion Checklist | | |
| Currently enrolled in an RCT for an intervention to reduce post-operative surgical site infection? *Tick one* | No | Yes |
| Current Body Mass Index (BMI) less than or equal to 18kg/m^2^? *Tick one* | No | Yes |
| Current Body Mass Index (BMI) greater than or equal to 45kg/m^2^? *Tick one* | No | Yes |
| Has Diabetes (type 1, Type 2 or gestation)? *Tick one* | No | Yes |
| Has pregnancy induced hypertension? *Tick one* | No | Yes |
| Has a Cardiovascular Disease (eg. Maternal structural cardiac disease)? *Tick one* | No | Yes |
| Has Renal disease?  *Tick one* | No | Yes |
| Has Liver disease? *Tick one* | No | Yes |
| Has suspected pre-existing infection (including Chorioamnionitis)? *Tick one* | No | Yes |
| Has received an antibiotic in the past 1 week prior to delivery? | No | Yes |
| Please note: if any of the shaded boxes above are ticked, then the woman is ineligible to take part in this study. Please sign and date the form. | | |

| **Section 4 - Form Completion Details** | | |
| --- | --- | --- |
| Full name of person completing form: | Signature of person completing form: | Date:  **D D** - **M M M** - **Y Y Y Y** |

C-LACE Operation Form

| Section 1 - Participant Details |
| --- |
| Participant Study ID Number:   \|  \|  \|  \|  \|  \| \| --- \| --- \| --- \| --- \| --- \| |

| Section 2 – Current weight and height | | |
| --- | --- | --- |
| Current Weight: **.** kg | Height: cm | Gestational age in weeks at time of CS Weeks |

| Section 3: Current medication within the last 48 hours prior to CS: | | | | | | | |
| --- | --- | --- | --- | --- | --- | --- | --- |
|  | **Drug Name/ Strength** e.g: | **Dose** | **Frequency** | **Route** | **Starting Date** | **Discontinuation date** | **Comments** |
| 01 |  |  |  |  | **D D** - **M M M** - **Y Y Y Y** | **D D** - **M M M** - **Y Y Y Y** |  |
| 02 |  |  |  |  | **D D** - **M M M** - **Y Y Y Y** | **D D** - **M M M** - **Y Y Y Y** |  |
| 03 |  |  |  |  | **D D** - **M M M** - **Y Y Y Y** | **D D** - **M M M** - **Y Y Y Y** |  |
| 04 |  |  |  |  | **D D** - **M M M** - **Y Y Y Y** | **D D** - **M M M** - **Y Y Y Y** | If more than 4 medicines, complete this table and tick this box *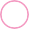* |

| Section 4 - Details of the Caesarean Section | | |
| --- | --- | --- |
| Was the Caesaran section performed in labour? *Tick one 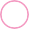* No 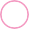 Yes | | If **YES**, how many cm dilated? cm |
| Did the woman have rupture of membranes before the caesarean section? *Tick one 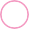* No 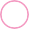 Yes | | |
| If **YES**, date of rupture of membranes: **D D** - **M M M** - **Y Y Y Y** | Time of rupture of membranes: *24hr* **H H. M M** | |
| Type of anaesthesia *Tick one 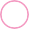* General 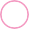 Regional | Time of administration of anesthesia *24hr* **H H. M M** | |
| Any additional fluid administered *e.g. IV isotonic fluid***?** *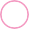* No 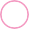 Yes | Type of the fluid :*g. IV isotonic fluid* | |
| Estimated fluid administrated: mls | Estimated blood loss: mls | |

| Section 5: Prophylactic antibiotic administration: | | | | | | | |
| --- | --- | --- | --- | --- | --- | --- | --- |
|  | **Drug Name**:  *e.g Cefuroxime* | **Dose** :  *e.g 1500 mg* | **Frequency**  *e.g once* | **Route**  *e.g IV* | **Exact time of antibiotic given** *24 h* | **Timing from administration to knife to skin incision** *e.g 30 min prior Skin incision or 60 min post Skin closure* | **Comments** |
| 01 |  |  |  |  | **H H** - **M M** |  |  |
| 02 |  |  |  |  | **H H** - **M M** |  |  |
| 03 |  |  |  |  | **H H** - **M M** |  |  |
| 04 |  |  |  |  | **H H** - **M M** |  | If more than 4 antibiotics, complete this table and tick this box *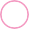* |

| Section 6 - Operation Details | |
| --- | --- |
| Date of CS decision: *e.g. 31-JAN-2019* **D D** - **M M M** - **Y Y Y Y** | Time of CS decision: *24 h* **H H** - **M M** |
| Date of knife to skin: *e.g. 31-JAN-2019* **D D** - **M M M** - **Y Y Y Y** | Time knife to skin: *24 h* **H H** - **M M** |
| Date of knife to uterus: *e.g. 31-JAN-2019* **D D** - **M M M** - **Y Y Y Y** | Time of knife to uterus: *24 h* **H H** - **M M** |
| Date baby delivered: *e.g. 31-JAN-2019* **D D** - **M M M** - **Y Y Y Y** | Time baby delivered: *24 h* **H H** - **M M** |
| Date placenta delivered: *e.g. 31-JAN-2019* **D D** - **M M M** - **Y Y Y Y** | Time placenta delivered:*24 h* **H H** - **M M** |
| Date Skin Closure: *e.g. 31-JAN-2019* **D D** - **M M M** - **Y Y Y Y** | Time skin closure skin: *24 h* **H H** - **M M** |
| Date woman out of theatre: *e.g. 31-JAN-2019* **D D** - **M M M** - **Y Y Y Y** | Time out pf theatre: *24 h* **H H** - **M M** |
| Abdominal incision *Tick one 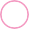* Midline *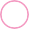* Transverse | Thickness of adipose tissue cm *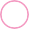* Estimate *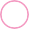* Measured |
| Uterine incision: *Tick one 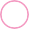* Lower segment *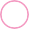* Classical *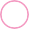* T shaped *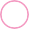* Extension into upper segment | |

| Section 7 - Details of samples collected | | | | | |
| --- | --- | --- | --- | --- | --- |
| Sample number | Type of sample | Site of fat tissue collected | Date taken *e.g. 31-JAN-2019* | Time taken *24 h* | Time within CS *Tick one* |
| 1 | 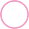 Blood sample |  | **D D** - **M M M** - **Y Y Y Y** | **H H** - **M M** | 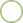 at time of skin incision  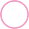 at time of skin closure |
| 2 | 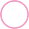 Blood sample |  | **D D** - **M M M** - **Y Y Y Y** | **H H** - **M M** | 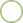 at time of skin incision  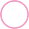 at time of skin closure |
| 2 | 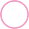 Blood sample |  | **D D** - **M M M** - **Y Y Y Y** | **H H** - **M M** | 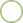 during recovery |
| 3 | 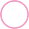 adipose tissue sample | 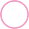 upper segment of fat layer  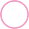 middle segment of fat layer  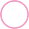 lower segment of fat layer | **D D** - **M M M** - **Y Y Y Y** | **H H** - **M M** | 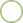 at time of skin incision  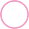 at time of skin closure |
| 4 | 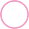 adipose tissue sample | 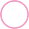 upper segment of fat layer  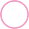 middle segment of fat layer  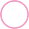 lower segment of fat layer | **D D** - **M M M** - **Y Y Y Y** | **H H** - **M M** | 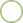 at time of skin incision  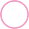 at time of skin closure |

| **Section 10 - Form Completion Details** | | |
| --- | --- | --- |
| Full name of person completing form: | Signature of person completing form: | Date: *e.g. 31-JAN-2019*  **D D** - **M M M** - **Y Y Y Y** |

C-LACE Trial Discharge

| Section 1 - Participant Details |
| --- |
| Patient trial No:   \|  \|  \|  \|  \|  \| \| --- \| --- \| --- \| --- \| --- \| |

| Section 2 - Discharge Details | | |
| --- | --- | --- |
| Discharge date: *e.g. 31-JAN-2017* **D D** - **M M M** - **Y Y Y Y** | Discharge time: | *24hr* **H** **H** *.* **M** **M** |

| Section 3 - Antibiotics | | | | | | | |
| --- | --- | --- | --- | --- | --- | --- | --- |
| Was the woman prescribed a course of antibiotics postnatally? *Tick one* 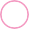 No 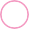 Yes | | | | | | | |
| If **No**, please go to Section 4, If **YES**, complete the following regarding antibiotics given postnatally | | | | | | | |
|  | **Drug Name/ Strength** e.g: *e.g Cefuroxime* | **Dose** *e.g 1500 mg* | **Frequency** *e.g twice* | **Route** *e.g. IV* | **Starting Date** *e.g. 31-JAN-2019* | **Discontinuation date** *e.g. 31-JAN-2019* | **Comments** |
| **1** |  |  |  |  | **D D** - **M M M** - **Y Y Y Y** | **D D** - **M M M** - **Y Y Y Y** |  |
| **2** |  |  |  |  | **D D** - **M M M** - **Y Y Y Y** | **D D** - **M M M** - **Y Y Y Y** | If more than 2 antibiotic, complete this table and tick this box *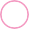* |
| Was the woman discharged on antibiotics? *Tick one* 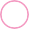 No 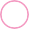 Yes | | | | | | | |
| Were the antibiotics used for a suspected/confirmed surgical site infection (e.g. uterine, pelvic, abdominal wound, perineal)? *Tick one*  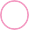 No 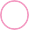 Yes | | | | | | | |
| If the discharge antibiotic are as reported in table above, please go to section 4; if the discharge antibiotic different from reported antibiotic in table above, then complete this table regarding discharge antibiotic | | | | | | | |
|  | **Drug Name/ Strength** e.g: *e.g Cefuroxime* | **Dose** *e.g 1500 mg* | **Frequency** *e.g twice* | **Route** *e.g. IV* | **Starting Date** *e.g. 31-JAN-2019* | **Discontinuation date** *e.g. 31-JAN-2019* | **Comments** |
| **1** |  |  |  |  | **D D** - **M M M** - **Y Y Y Y** | **D D** - **M M M** - **Y Y Y Y** |  |
| **2** |  |  |  |  | **D D** - **M M M** - **Y Y Y Y** | **D D** - **M M M** - **Y Y Y Y** | If more than 5 antibiotic, complete this table and tick this box *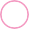* |

| Section 4 - Suspected or Confirmed Infection | |
| --- | --- |
| Did the woman develop a fever **(≥38°C)** during her inpatient stay? *Tick one 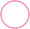* No; Temp <38°C 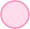 Yes; Temp ≥38°C | |
| Was an infection suspected and/or confirmed in the uterus/pelvis? *Tick one*  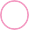 No 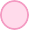 Yes | If **YES**, was infection prompted by maternal and/or clinical concern? *Tick one*  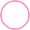 Maternal concern 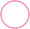 Clinical concern 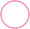 Both |
|  |  |
| Was an infection suspected and/or confirmed in the abdominal wound? *Tick one*  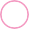 No 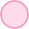 Yes | If **YES**, was infection prompted by maternal and/or clinical concern? *Tick one*  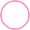 Maternal concern 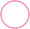 Clinical concern 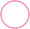 Both |
|  |  |
| Was an infection suspected and/or confirmed in the perineum? *Tick one*  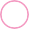 No 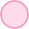 Yes | If **YES**, was infection prompted by maternal and/or clinical concern? *Tick one*  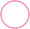 Maternal concern 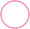 Clinical concern 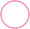 Both |
|  |  |

If **any shaded box is ticked in section 4**, please complete rest of form. If not, please sign and date the form

| Section 5 - Signs & Symptoms | |
| --- | --- |
| Did the woman have any of the following? *Answer all* | |
| Abnormal uterine tenderness 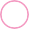 No 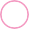 Yes | Abdominal wound tenderness 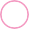 No 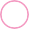 Yes |
| Abnormal uterine involution 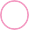 No 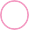 Yes | Abnormal lochia No Yes |
| Abnormal temperature No Yes | |
| Did the woman have a clinical diagnosis of endometritis requiring treatment? *Tick one*  No Yes | |
| If **YES**, date of clinical diagnosis of endometritis: *e.g. 31-JAN-2017* **D D** - **M M M** - **Y Y Y Y** | |
| Did the woman meet the **CDC definition of endometritis** below? (see CDC definition) *Tick one*  No Yes | |
| The definition as per the CDC are: Patient has organisms cultured from endometrial fluid or tissue (including amniotic fluid) OR Patient has at least two of the following: fever (≥38°C), pain or tenderness (uterine or abdominal), or purulent drainage from uterus (day 0 - 30) | |

| Section 6 - Microbiology | |
| --- | --- |
| Did the woman have any microbiology samples taken post operatively? *Tick one*  No Yes | |
| If **No**, go to Section 7 | |
| Was a **blood culture** sample taken? *Tick one*  No Yes | |
| If **YES**, did the **blood cultures** grow any micro-organisms? *Tick one*  No Yes | If **YES**, please specify the micro-organisms: |
| Was a **skin wound swab** sample taken? *Tick one*  No Yes | |
| If **YES**, did the **skin wound swab** grow any micro-organisms? *Tick one*  No Yes | If **YES**, please specify the micro-organisms: |
| Was a **vaginal swab** sample taken? *Tick one*  No Yes | |
| If **YES**, did the **vaginal swab** grow any micro-organisms? *Tick one*  No Yes | If **YES**, please specify the micro-organisms: |
| Was an **MSU** sample taken? *Tick one*  No Yes | |
| If **YES**, did the **MSU** grow any micro-organisms? *Tick one*  No Yes | If **YES**, please specify the micro-organisms: |
| If **any other** samples were taken, please specify site and micro-organism if positive culture: | |

| Section 7 - Severity of Infection |
| --- |
| Was the woman admitted to level 2 or level 3 care (e.g. HDU) as a result of a suspected/confirmed infection? *Tick one*  No Yes |
| Did the woman develop sepsis prior to discharge from hospital? *Tick one*  No Yes |
| If **YES**, date of diagnosis of sepsis: *e.g. 31-JAN-2017*  **D D** - **M M M** - **Y Y Y Y** |
| Did the infection delay the woman's discharge from hospital? *Tick one*  No Yes |
